# Looking beyond the individual–The importance of accessing health and cultural services for Indigenous women in Thunder Bay, Ontario

**DOI:** 10.1371/journal.pone.0282484

**Published:** 2023-03-01

**Authors:** Jonathan C. Lin, Elaine Toombs, Chris Sanders, Candida Sinoway, Marni Amirault, Christopher J. Mushquash, Linda Barkman, Melissa Deschamps, Meghan Young, Holly Gauvin, Anita C. Benoit

**Affiliations:** 1 Dalla Lana School of Public Health, University of Toronto, Toronto, Ontario, Canada; 2 Lakehead University, Thunder Bay, Ontario, Canada; 3 Elevate NWO, Thunder Bay, Ontario, Canada; 4 Communities, Alliances & Networks, Dartmouth, Nova Scotia, Canada; 5 Ontario Aboriginal HIV/AIDS Strategy, Thunder Bay, Ontario, Canada; 6 Ontario Aboriginal HIV/AIDS Strategy, Toronto, Ontario, Canada; 7 Women’s College Research Institute, Women’s College Hospital, Toronto, Ontario, Canada; 8 Department of Health and Society, University of Toronto Scarborough, Scarborough, Ontario, Canada; National Institute of Public Health, MEXICO

## Abstract

Access to cultural activities and culturally relevant healthcare has always been significant for achieving holistic Indigenous health and continues to be a key factor in shaping the health journey of Indigenous individuals and communities. Previous research has indicated the importance of cultural practices and services in sustaining cultural identity for Indigenous peoples, which is a major influence on their wellbeing. This study marks the first phase in a project aimed at establishing an Indigenous healing program and uses a qualitative research approach to understand the health and cultural services that Indigenous women want and require in Thunder Bay, Ontario. During interviews, participants (n = 22) answered questions around their understandings of health and wellbeing, and how they are able to incorporate cultural practices into their circle of care. Thematic analysis was performed on interview transcripts, and 4 key themes were identified: ‘independence and self-care’, ‘external barriers to accessing services’, ‘finding comfort in the familiar’ and ‘sense of community’. Together these themes illustrate how Indigenous women feel a strong sense of personal responsibility for maintaining their health despite the multiple environmental factors that may act as barriers or supports. Furthermore, the necessity of embedding cultural practices into Indigenous women’s circle of care is highlighted by the participants as they describe the mental, spiritual, social, and emotional health benefits of engaging in cultural activities within their community. The findings demonstrate the need for current modes of care to look beyond the individual and consider the impacts that socio-environmental factors have on Indigenous women. To accomplish this, we hope to increase access to health and cultural services through the creation of an Indigenous healing program that can be adequately incorporated into Indigenous women’s circle of care if they wish to do so.

## Introduction

Cultural continuity is the level of social and cultural cohesion within a community, particularly involving intergenerational connectedness, which is maintained through engagement with family members or other community members that transmit knowledge and pass on traditions to subsequent generations [1, 2]. Cultural continuity has been identified as a determinant of health for Indigenous Peoples, and is associated with positive health outcomes that are achieved through ceremonies, intergenerational transmission of cultural knowledge, and creating a sense of belonging and cultural identity [1, 3]. Cultural identity acts as a major influence on improving Indigenous girls’ and women’s confidence and self-esteem by dispelling common negative stereotypes or assumptions attached to them by society [4]. Having this strong sense of cultural identity and self-esteem has been linked with positive outcomes for wellbeing by playing a protective role for mental and emotional health in Indigenous populations, particularly Indigenous youth [3, 5–8].

Indigenous peoples have long engaged in their cultural practices as part of healing and medicine, sharing teachings, and maintaining reciprocal relationships with the environment [9–12]. Ensuring culture-based treatment options for Indigenous service users has the potential to influence broader wellbeing for Indigenous peoples, as self-care is largely embedded within cultural practices such as smudging, ceremony, and the gathering and use of medicinal plants [13, 14]. There is a diverse range of culturally-based practices and treatments for Indigenous peoples, which derive from a variety of Indigenous traditions that exist among different Indigenous groups [14]. It is necessary to support continued access to these cultural practices and healing approaches for Indigenous women in their circle of care, as maintaining these practices also help to improve autonomy and access to diverse health services [15].

Indigenous peoples continue to face various barriers in obtaining adequate and equitable health care in Canada [16]. This has been partially attributed to the emphasis on predominately non-Indigenous health services of biomedical paradigms that are unable to encapsulate the unique needs and experiences of Indigenous Peoples fully [17–19]. Previous studies have shown that health care provisioning can be improved when the model of care is tailored to the needs of, or owned and managed by Indigenous communities themselves [20, 21]. Having the autonomy to control and decide what services they want is likely to lead to a model of health care provisioning that minimizes experiences of discrimination for Indigenous Peoples while maximizing cultural relevance [3, 22].

Beyond Indigenous healing approaches, Indigenous health and wellbeing also signifies the importance of relationships and interdependence among individuals and their families, communities, nature, and spirit [23, 24]. Having support from relationships and the wider community have been associated with thriving health for Indigenous individuals, and it has been suggested that health programs may have greater health effects if they simultaneously build on supporting positive social interactions across the community level [25, 26]. Both service users and service providers must understand that many socio-environmental factors play a role in holistically shaping an individual’s health, and self-care practices are only part of the greater whole. Maintaining relationships with land, nature, and the social and cultural environment is a critical component in achieving holistic health for Indigenous Peoples, and acknowledging this in mainstream services will be key in expanding the relevance and acceptability of services for Indigenous women while minimizing instances of self-blame for those experiencing poor health outcomes [25, 27–29]. This paper will describe four key themes identified from participant interviews that illustrate the importance of considering the multiple actors involved in influencing an individual’s ability to access health and cultural services for their wellbeing. We then conclude by discussing how these themes may be useful when informing or conceptualizing the creation of an Indigenous healing program.

This study describes the first phase in a project aimed at establishing an Indigenous healing program that is sustainable and accessible for Indigenous women living in Thunder Bay, Ontario. The study objectives were to determine Indigenous women’s understanding of health and wellbeing, and to identify Indigenous healing approaches that they use or desire. Findings will inform the development of the Indigenous healing program.

## Methods

### Research team

Our community partners were Elevate NWO and the Ontario Aboriginal HIV/AIDS Strategy (Oahas). Elevate NWO is taking action to address the Truth and Reconciliation Committee of Canada’s (TRCC) [30] Call to Action #22: “We call upon those who can effect change within the Canadian health-care system to recognize the value of Aboriginal healing practices and use them in the treatment of Aboriginal patients in collaboration with Aboriginal healers and Elders where requested by Aboriginal patients.” Elevate now is a support service in Northwestern Ontario that provides counselling and referrals regarding issues related to HIV/AIDS, hepatitis C, and harm reduction information or supplies. They are focused on prevention, community outreach, advocacy, and case management, and are committed to the TRC Calls to Action across all their work. The Indigenous healing program resulting from this study will be housed at Elevate NWO and be made available to its clients. In addition, it will also be available to clients accessing services from the Oahas, which is based within Elevate NWO. Oahas delivers culturally grounded programs and services including harm reduction to prevent the transmission of HIV and other sexually transmitted blood borne infections. They closely collaborate with Elevate NWO in research and the delivery of services. Members of both organizations are represented on our research team (CAS, LB, MY, MD, HG). Moreover, our research team included First Nations individuals as service providers, academic and community researchers.

Five of the 11 co-authors are First Nations (CAS, CJM, LB, MY, MD) with one author being First Nations and French Acadian (ACB). Several of the authors (ET, CAS, CJM, LB, MY, MD, HG, MA) work in and/or lead organizations which provides health, social and cultural services to Indigenous people. Five authors are academic researchers (JCL, ET, CS, CM, ACB) and 6 are community researchers (CAS, MA, LB, MY, MD, HG). Three authors (JCL, CAS, LB) were hired to work on the research project. All authors are experienced in community-based research (CBR). Seven authors (ET, CHS, CAS, CM, LB, MD, HG) call Thunder Bay home and one co-author (MY) is the executive director of an organization that has several regional sites including Thunder Bay.

### Two-eyed seeing guiding principles and community-based research

The research process for this project is guided by Etuaptmumk, or Two-Eyed Seeing, which means “learning to see from one eye with the strengths of Indigenous knowledge and ways of knowing, and from the other eye with the strengths of western knowledges and ways of knowing, and to use both these eyes together for the benefit of all” [31]. Beyond acknowledging different types of perspectives, deeper premises of Etuaptmumk involve inclusion of spiritual knowledge in human understandings of the world, co-learning processes, and considerations on the value and impact of our current actions for seven generations ahead [31–33]. Using the Two-Eyed Seeing guiding principle enables the strengths of research team members as well as study participants to be harnessed, and further reinforces the project to be shaped by diverse perspectives that include the knowledges of both Indigenous and allied stakeholders. Two-Eyed Seeing was implemented during data collection to allow a safe space for participants to speak about their lived experiences openly, without fear of providing “wrong answers” or being subject to judgement from the interviewers. To be able to see multiple perspectives, it is important to be reflexive of the diverse knowledges held in the research setting. Active reflexivity allows researchers to continually practice critical self-awareness throughout the research process, helping to enhance both the integrity of research as well as the quality of knowledge produced [34].

Further, a CBR approach was followed, which inherently values multiple ways of knowing, is collaborative, and equitably involves all research team members who choose how they would like to participate [35]. The conceptualization of the study had originated from our partner organization Elevate NWO to address the TRCC’s calls to action #22 and support the agenda of their long-time collaborator Oahas, another partner, as well as respond to the needs of their Indigenous clients. Also, various community member and knowledge user perspectives who were part of the research team contributed to conceptualizing the study. Using the CRediT methodology, we have outlined the research team members contributions as authors. Prior to implementation of any study activities, the research team consulted with relevant stakeholders including community leaders and individuals with lived experience to participate in the development of the socio-demographic questionnaire and interview guide. We hired local Indigenous persons as research staff (i.e., Elder, research assistant) who became part of the research team. Our research staff recruited study participants, led the data collection process (e.g., informed consent, administering questionnaire, interviews) and participated in data analysis to prepare this manuscript. Our student research assistant took the lead on developing the codebook from the interview transcripts. The student held research team analysis meetings to review the codebook and analyze the transcripts. They prepared the draft manuscript, circulated it for feedback and incorporated their feedback to finalize the submitted manuscript.

### Participant sampling and recruitment

The study used convenience and snowball sampling to recruit potential participants. Recruitment flyers were posted, and cards were distributed by the community partners as part of the recruitment process in Thunder Bay, Ontario. Eligible participants were those who spoke and read in English, self-identified as First Nations, Métis, and/or Inuk (i.e., Indigenous), self-identified as a woman (including transgender women) and were aged 16 years or over at the time of data collection. Participants were deemed eligible if they initially endorsed that they needed or wanted to access cultural services (e.g., ceremonies, meeting with Elders) and emergency services (e.g., food banks, crisis response services) during the recruitment phase.

The eligibility criteria were chosen to reflect the demographic who can theoretically access the Indigenous healing program because they qualify for services from our community partner, and as such, included those living with HIV, hepatitis C, or in need of emergency services. Thus, we selected study participants who could qualify for these services to best inform the Indigenous healing program.

### Study consent

Each eligible participant provided both verbal and written informed consent prior to beginning research activities. Verbal consent was obtained using the online platform Zoom or over the phone and written consent was obtained by delivering the consent form through mail, email or during the in-person meeting. Ongoing consent is required by the research ethics board and was obtained verbally throughout the research. Our study received ethics clearance from Women’s College Hospital Research Ethics Board at Women’s College Hospital and the University of Toronto Research Ethics Board in Toronto, Ontario.

### Data collection

Data collection occurred during the COVID-19 pandemic. Canadian public health guidelines were followed as well as research ethics requirements related to return to in-person research. Screening for eligible participants was done over Zoom or the phone. Following screening, participants could complete the questionnaire and interview over Zoom, the phone or in-person. In-person data collection activities took place in a well-ventilated room at the community partner site and personal protective equipment (e.g., surgical masks, face shields, and hand sanitizer) was provided. Plexiglass separated the participants from the interviewer who were sitting face-to-face. Participants were provided with an individually wrapped refreshment and a drink during the interview.

#### Sociodemographic questionnaire

Participants completed a 10-minute sociodemographic questionnaire, which also asked individuals to describe their housing and sleeping situations, caregiving responsibilities, ongoing access to a health service provider or site, and where they go to access different types of health services. The interviewer used a tablet pre-loaded with the questionnaire in Qualtrics™ (Provo, UT).

#### Semi-structured interviews

Thirty-minute interviews took place between September 2020 –June 2021. A semi-structured interview guide was used to facilitate participant interviews. The focus of these interviews was primarily how participants understood their own individual health and wellbeing, how participants stay healthy, including how they apply Indigenous cultural practices for their health, and how participants would measure if something was working for their health. Some examples of interview questions included:

*“What is your ideal health journey?”*

*“What do you do to make yourself feel or be healthy?”*

*“What type of cultural things like ceremonies*, *teachings*, *or other cultural activities you like or would like to access to make yourself feel or be healthy*?”

Participants were encouraged to share as much detail as they were willing to when discussing these themes. Discussions were audio-recorded and transcribed.

#### Data analysis

Data were analyzed using thematic analysis, which consisted of coding the transcripts line by line to formulate descriptive themes [36]. Each transcript was read through once and initial codes of interest were highlighted by several research team members. Once recurring themes and patterns began to emerge, a second reading of each transcript was done by the research assistant to map out how coded lines with commonalities could be grouped under the same theme. Generated codes under the same theme were then added to a codebook and defined, and direct quotes were pulled from transcripts during a third reading to be included as examples of each code. The coding process occurred through a collaborative approach, with discussions between research team members on potential themes and key points of interest.

### Inclusivity in global research

Additional information regarding the ethical, cultural, and scientific considerations specific to inclusivity in global research is included in S1 Checklist.

## Results

### Participant characteristics

Thirty-four participants responded to the demographic questionnaire. The median age of participants at interview was 39.5 years [IQR: 31–44] (S2 Table). Participant characteristics are described in Table 1.

**Table 1:** Demographic characteristics of participants (n = 34).

Variable		N
Age (years)		
	<30	5
	30–49	24
	≥50	5
Living situation		
	Sleep and spend majority of time in same spaces	29
	Sleep and spend majority of time in different spaces	5
Caregiver responsibilities^1^		
	Yes	9
	No	24
	Prefer not to answer	1
Ongoing access to a healthcare provider		
	Yes^2^	29
	Doctor	23
	Nurse	14
	Social worker or counselor	15
	Other^3^	8
	No	5
Ongoing access to healthcare sites		
	Yes^4^	26
	Community health centre	17
	Emergency room	8
	Other^5^	16
	No	8
Chronic health conditions^6^		
	Hepatitis C	13
	Depression	23
	Anxiety	23
	Other^7^	20

^1^ Caregiver responsibilities include parents, children aged 16 and under (birthed), children aged 16 and under (kinship or other than birthed).

^2^ In some instances, participants listed accessing multiple healthcare providers. Each provider listed was counted as a unique response and are not mutually exclusive.

^3^Other healthcare providers mentioned include dentists, knowledge carriers (i.e., Elders, healers), and psychologists and psychiatrists.

^4^ In some instances, participants listed accessing multiple healthcare sites. Each provider listed was counted as a unique response and are not mutually exclusive.

^5^ Other healthcare sites mentioned include outreach clinics, walk-in clinics, Indigenous health centres, dental clinics, and hospitals.

^6^ In some instances, participants listed experiencing several chronic health conditions. Each chronic health condition listed was counted as a unique response and are not mutually exclusive.

^7^ Other chronic health conditions mentioned include addictions, arthritis, asthma, epilepsy, cancer, diabetes, heart disease, HIV, and post-traumatic stress disorder.

### Interviews

Of the 34 respondents who were screened and completed the questionnaire, 22 participants were interviewed to reach data saturation. Eighteen participated in one-on-one interviews resulting in 271 pages of transcripts. Four participants participated in groups of two with one interviewer, resulting in 38 pages of transcripts. A total of 309 pages of transcribed audio-recordings were read through as part of the thematic analysis process. After thematic analysis was completed, 10 total subthemes were identified, and were grouped into 4 separate composite themes. These composite themes were: (1) independence and self-care; (2) external barriers to accessing services; (3) sense of community; and (4) finding comfort in the familiar. ‘Independence and self-care’ were found to be a stand-alone theme with no subthemes grouped within it. Subthemes grouped under ‘external barriers to accessing services’ include (i) distance and transportation; and (ii) COVID-19. Subthemes grouped under ‘finding comfort in the familiar’ include (i) personal relationships; (ii) connection to land and nature; and (iii) demonstrated interest in culturally relevant activities. Finally, subthemes grouped under ‘sense of community’ include (i) knowledge and learning; and (ii) gathering. The first theme illustrates the Indigenous women’s understanding that their health status is indicative of their (in)ability to take care of themselves and make independent, healthy choices. The second theme contrasts with the first by spotlighting external factors that often act as barriers outside of Indigenous women’s control. The third and fourth theme together are sources of strength and familiarity that allow women to overcome socio-environmental barriers in accessing services by providing a sense of comfort and hopefulness. The total number of counted quotations representing each theme or subtheme are summarized in S1 Table.

**Theme 1: Independence and self-care.** Self-reliance and independence were associated with health status by participants. Several of the Indigenous women described that they needed to hold themselves responsible for their own health, and that the onus was on themselves when it comes to maintaining and protecting their wellbeing. This belief resulted in participants vocalizing that poor health outcomes were a result of an individual’s incapacity to manage their own health.

*I guess it’d be up to you*, *you’d probably be your own barrier if you can’t… if you want to change you have to do it for yourself*. *(P28*)

*Being unhealthy*, *you’re not taking care of yourself*. *You’re not balanced*, *like no self-care*. *You don’t really care about yourself*. *(P31*)

While feelings of self-blame and discouragement resulted from this belief that each individual holds responsibility over their health, participants also suggest feeling optimistic when they can listen to their minds and bodies to make health-conscious decisions. Being able to tune in to what your body and mind are telling you was described by a participant as helpful towards understanding when or how to practice self-care.

*Two nights ago*, *I was feeling like I wasn’t doing enough for myself and then I was saying I should take a long walk*. *I went to walk from my place to Shoppers and grabbed a bottle of water*. *And it felt real good*. *(P17*)

The participant perspectives highlight the importance of taking an active role in one’s wellbeing. These values and beliefs prove significant in the following sections when the women discuss both the external factors and structural forces that limit their ability to freely exercise decision-making power for their wellbeing.

#### Theme 2: External barriers to accessing services

Although the women believed they are responsible for their own health and must act independently in achieving good health, there are several barriers that may prevent them from doing so. The basis of this theme is on external factors identified by participants that prevent or deter them from being able to readily access health or cultural services. In this context, external barriers to accessing services are socio-economic or environmental factors that are often beyond individual’s control (e.g., availability of transportation, the ongoing COVID-19 pandemic). Being able to physically attend health and cultural services in-person was considered important by participants to their overall wellbeing, particularly when these services are convenient to access. These external barriers identified by the women demonstrate that despite their independence and responsibility in managing their own health, there are other challenges present that make it more difficult to maintain good health status.

*Distance and transportation*. The ability to both physically and financially access appropriate health services is widely acknowledged as a determinant of health [37, 38]. Areas where health services are located compared to participants’ households were identified as a barrier to physically accessing services. In conjunction, lacking methods of convenient transportation exacerbated the problem of geographically distant health services. For the women, this included lacking a car, or the added costs of purchasing bus passes every month.

*I had trouble getting to my appointments to see my therapist before because I don’t have a car and it’s in this really weird spot to take the bus*. *I even have to walk a distance*. *I would have to hike from the bus stop to the doctor*. *(P5*)

One of the women mention that knowing what services to access aren’t the problem; rather, being able to physically reach them is the challenging part. She mentions how walking is not a suitable option for her personally, as she has limited capacity for physical movement due to other pre-existing health issues.

*I know there are services*. *But you know what*, *how do I get to the services is the issue*. *Before my doctor retired a couple of years ago*, *he told me if it’s winter*, *do not walk anywhere*. *He says*, *because if you slip and fall*, *you might not walk again*. *(P44*)

*COVID-19*. At the time the interviews were conducted, lockdowns due to the coronavirus (COVID-19) pandemic were occurring across Canada. Public health regulations such as physical and social distancing, as well as bans on gatherings, were cited by research participants as obstacles to fully engaging in social and cultural activities to stay healthy. Public health messaging at the time was also strongly recommending Indigenous individuals to halt participation in ceremonies, due to the potential for COVID-19 infection [39]. These regulations were a main source of dissension and frustration among the Indigenous women, as they described the many ways COVID-19 was impacting their ability to connect with family, friends, and other community members.

*Sometimes I say I’m sick and tired of this COVID*, *even though I’m not sick with COVID*. *Like I want my ceremonies back*. *I want to do the gatherings we used to do*. *The get-togethers*. *And learning culture*. *And doing workshops*. *And doing Indigenous things that we did like root picking*, *sweetgrass picking*, *and all that*. *(P13*)

*I’ve been neglecting my health*, *by not taking my meds*. *By not smudging*. *No sweats*. *It’s like everything is low… with COVID you have to stay indoors right*. *So*, *you can’t really go out and about*. *(P17*)

Because of COVID-19 restrictions on gatherings, participants vocalized feeling a decline in their overall wellbeing not due to contracting the disease itself, but because of their inability to partake in activities for their health. These activities included going to the gym, sage picking, and moose hunting–all of which participants demonstrated having a strong desire to engage in. Similarly, transitioning to virtual methods of health care delivery (e.g., telemedicine, phone calls) was a challenge for some participants, with one particular participant mentioning that she discontinued sessions with her therapist as it felt uncomfortable and less personal to describe her problems over the phone. The impacts to social wellbeing are highlighted by a participant who describes how COVID-19 has “isolated a lot of people” and because of this physical and social isolation, “people are not getting the necessary help that they need.”

#### Theme 3: Finding comfort in the familiar

This theme focuses on participants describing how being in environments of familiarity or around familiar individuals elicit feelings of comfort and closeness. These settings allowed individuals to be at ease and express themselves openly, which was mentioned to be ideal for health and wellbeing. Participants drew on familiarity in their physical and social environments, using these facets of their life as sources of strength to overcome burdens on their health.

*Personal relationships*. A commonly identified factor that acts as a facilitator for good health was the connections that each Indigenous woman had with those closest to them–friends, family, colleagues, Elders, and other confidants. Many participants described their relationships with close friends or family members as being key components to feeling or staying well, sometimes relating these emotional bonds as ways to uplift their mood or provide motivation to get through the day.

*I had my grandparents and my kids and all*. *So*, *I rarely drank and stuff*. *Yeah*, *I always had my family around me*. *And it made me happy all the time*. *I wasn’t depressed*, *I wasn’t having anxiety*. *Yeah*, *it was just… that was fun*. *(P34*)

*I have access to my Elder here and I’m grateful for having her here when she can be here*. *I think I would feel lost if I never got to see her again*. *She makes me feel proud to be her friend*. *(P26/27*)

Relationships were acknowledged by participants as supporting networks that can “help each other,” and are meant to benefit both parties involved by looking out for one another where necessary. One participant mentions the potential for relationships to be harmful on the emotional and mental state, saying that they only “stay around positive people if [I] can. I try to stay away from the negative people that bring me down about their problems”. The majority of participants spoke positively about the relationships that they had constructed, and described how they continue to foster and maintain these relationships within their social circle. However, it is also important to note that in the case for one participant, relationships were described as a burden to her health. The participant describes feeling uncomfortable at home due to her controlling boyfriend and having a lack of autonomy to do or say what she wants around him:

*My relationship gets in the way because he’s a little bit controlling… if you’re fighting with somebody constantly*, *I think it’s lowered my immune system a little bit*. *I think that might have had something to do with it*. *(P5*)

*Connection to land and nature*. Maintaining a closeness to nature and the land was described by participants as a comforting feeling and a major contributor to overall wellbeing. In combination, engaging in recreational activities outdoors were strongly appreciated and allowed participants to feel connected to the natural world.

*Being in the Bush*, *especially in the springtime when everything is just coming alive again*, *and then it’s blooming and you can smell all the medicines*. *It’s almost like you want to inhale all of that… then you’re close by you know*. *It’s there*. *It just makes you feel well*. *(P13*)

*I guess being one with the Earth right*. *I used to go and sit and like walk around the edges of the community*, *and like sit there and be alone and meditate*. *(P42*)

There was an association between participants’ connection to land and nature, and their interest for culturally relevant activities. Many of the women described being able to feel closer to their culture by being outside. Land and nature can act as outlets for the women to draw strength from while partaking in cultural activities, and some of the women also felt that being in nature improved their spiritual wellbeing. One woman described being spiritually healthy means being in the bush and having the “freedom of being in the wilderness.” Another woman echoed these thoughts more broadly, saying that to be spiritually healthy, she would have to feel “connected with the world around [her].”

*Demonstrated interest for culturally relevant activities*. Strong desires to participate in culturally appropriate and relevant activities were a focal point during the interviews. Participants mentioned various types of cultural activities and ceremonies that they were either interested in wanting to attempt for the first time or partaking in on a consistent basis. These included pow-wows, pipe ceremonies, cedar baths, ceremonial dancing, drum-making, drumming, beading, learning their native language, and singing.

*Last year I went on a trip with some ladies for a ceremony*. *We did the ceremonies*, *we brought all our sacred items and bundles*. *Then*, *we did drumming and went and looked at the plants and learned about the plants*. *It was fun*, *it was really good… I’d really*, *really like to do that again*. *(P5*)

I would love to go back to a sweat lodge. I used to love going in there. And I think the most powerful sweat lodge is when there’s lots of people in there. And you know I always hear how people can hear things in there, but I have never heard anything. Some people say they hear the eagle, and some people say they feel the bear paw, but I have never gone through any of that in a sweat lodge. And I always want to keep going because it’s relaxing when you come out of there too because I’ve let a lot of stuff go. I feel light when I come out. (P26/27)

*I would love to do another pipe ceremony*. *It was so awesome to help that time*. *To be able to hold the pipe*, *to take it apart*, *to put it the proper way*. *And to know all those teachings on how you take care of your equipment*. *(P43*)

The women vocalized the importance of connecting to culture by engaging in these activities as a positive contribution to their emotional state and wellbeing. One participant described that when they’re feeling down or depressed, they like to smudge to help clear the emotions going through their mind.

#### Theme 4: Sense of community

Indigenous women vocalized the importance of feeling a sense of community through social activities or ceremonies as another source of strength. This feeling of togetherness does not necessarily have to stem from traditional ceremonies like the sweat lodge but can be from informal and casual gatherings. The sentiment of being around other relatable individuals were highlighted by several participants as comforting and facilitates a safe space to share knowledge and stories.

*Knowledge and learning*. Sharing knowledge and lived experiences with others was one of the ways that Indigenous women described building a sense of community and the intergenerational transmission of knowledge. Passing on traditional teachings or stories was described by participants as a way of feeling healthier by connecting with others in the community.

*Teaching*. *I love telling stories*. *Sharing*. *That makes me feel healthy*. *Like when I walk out of the building ‘hey I did something’ like ‘oh wow*, *I still got it’ you know*. *(P17*)

*Knowing your practices and the teachings*. *And speaking to your elders regularly*, *seeking guidance*. *And then passing on your knowledge to others*. *(P41*)

*“I think a lot of Natives need to learn from the Elders before they die*. *We’re losing everything*. *Because after I pass away*, *my daughter is not going to know nothing*. *Like basically the government is succeeding in assimilating everyone*. *So*, *unless we change that*, *we’re not going to get our culture back or learn anything or have anything to teach our kids*. *We’re just going to mesh into nothing*.*” (P42*)

As well, the women themselves also expressed wanting to learn more about Indigenous practices and teachings from fellow community members. One woman described the importance of maintaining a cycle in knowledge sharing, mentioning that it is important to speak to Elders regularly to seek guidance and then to pass on that knowledge to others you know. When asked about what she does to feel healthy, another woman says:

*I go see my Elder*. *Learn from her what her teaching is*. *Just talking*. *It feels good after*. *(P29*)

*“I just need more Native culture*. *Understanding more*, *I got to learn to take care of myself*, *learn more education on my own health*. *An Elder showed me certain things to how to smudge and that*. *So*, *I’m slowly learning*.*” (P29*)

*Gathering*. The physical act of gathering around family, friends, or other familiar members in the community as a form of social connectedness was associated with good health by participants. This subtheme contrasts with the subtheme ‘*personal relationship*s’ because in this theme, participants emphasized the importance of being in a social setting with multiple people at one time. The Indigenous women described that while they may not have close personal relationships with each individual in settings such as get-togethers or sharing groups, they felt that the aspect of togetherness was beneficial to their mental and social health. More specifically, getting involved in social gatherings allowed women to feel included and a sense of belonging to social circles that they can relate to.

*Traditionally*, *I feel healthy when there used to be gatherings*. *Gathering together as a family*, *and not as strangers*. *(P26/27*)

*Plus*, *you know what*, *being involved with groups*, *going to… I can’t even think of the terms*. *Like what we’re doing now*. *Just being around people*. *Basically*, *a sharing group*. *Like even just sitting around the campfire*, *get-togethers*. *I don’t know*, *I just miss being around people*, *period*. *I think that’s what made me happy*, *that’s what makes me feel loved*, *that’s what I guess when I felt healthy*, *mentally*. *(P44*)

## Discussion

The study findings reveal that despite health and wellness being attributed to independence and self-reliance of participants, there are external factors beyond the individual that can impact their health condition–for better or worse. Thus, it is increasingly relevant for health service models to look beyond the individual, and to consider the contexts of the environment surrounding them; particularly for Indigenous Peoples, where cultural-relevance, social relationships, and connection to land are held so closely in line with wellbeing [1, 40].

The theme ‘independence and self-care’ exemplifies how Indigenous women associate being healthy with self-reliance. When asked about how they interpret meanings of health and wellbeing, participants set expectations for themselves in achieving good health and believe they must hold themselves accountable in reaching those expectations. Self-blame and having a sense of responsibility for one’s own wellbeing has been a pattern noticed in other areas of health, including chronic disease management [41–43]. This unyielding belief of independence in managing one’s own health status can be related to individualist ideologies in mainstream health services that are used to explicitly or implicitly place blame on Indigenous Peoples when poor health outcomes are observed [44–47]. Within the field of health promotion, there have been criticisms of focusing too much attention on individualistic and lifestyle factors, while not fully taking into consideration the environmental and contextual forces that may similarly influence an individual’s health journey [46–48]. Thus, the socio-ecological model has been used as an approach towards better encapsulating the interpersonal, community, and societal levels of influences on individual health [49]. For example, at the time this study was conducted, the two most prominent external factors identified as barriers included the ongoing COVID-19 pandemic and inconvenient distances or the lack of transportation to physically access services. Not only did the pandemic prevent Indigenous gatherings for social and cultural practices, it also exacerbated problems with healthcare access, particularly in contexts regarding testing and the shift to virtual modes of healthcare delivery [39]. This creates potential challenges for Indigenous individuals who may not have internet or cellular connections strong enough to support the demands of video or telephone exchanges with health service providers [39]. Although this problem specifically was not described by participants in the study, one participant mentions feeling that virtual methods of health care delivery felt less personal than in-person sessions and discontinued talking with her therapist as a result. Maintenance of therapeutic relationships and limited engagement between patient and provider have previously been cited as challenges in the shift towards virtual health care delivery methods [50–52].

Participants also cited that knowing where to access health services was not an issue, but being able to physically access them posed more of a challenge. Physical access to health services often is related to the geographical distance to services, or an individual’s ability to overcome the time and cost associated with transportation to the service [1, 37]. While physical distances and lack of transportation options were described by participants as barriers towards attending services, it is equally critical to consider the relevance of social distances between patient and provider. Reducing social distances in health care means establishing spaces that are safe, culturally-appropriate, and socially inviting [37, 53, 54]. Adopting this culturally-safe approach to providing care may encourage greater trust and willingness for Indigenous women to seek out health care workers for their health concerns, rather than avoiding health care workers under the fear of being discriminated against [39, 37, 53–55]. These barriers signify the relevance of considering external or environmental factors beyond the individual level as potential influences on poor health outcomes.

Despite the strengths of the socio-ecological model in acknowledging the interactions across individual, interpersonal, and societal levels, it does not fully apply to Indigenous communities due to the lack of acknowledging connections to culture, spirit, and land. As such, we propose referencing the First Nation Health Authority’s (FNHA) visual depiction of First Nations Perspective on Health and Wellness [40] as a more appropriate and relevant model in discussions on Indigenous wellbeing. The First Nations Perspective on Health and Wellness model includes considerations of environmental, cultural, economic, and social aspects of an individual in the most outer ring [40]. Other important determinants of health including knowledge, family, and land are included in the model as well [40], and are able to clearly represent the range of factors that influence health status for Indigenous Peoples compared to the standard socio-ecological model. This is reflected in this study’s findings, as the women described the importance of having supports from the wider community and natural world for achieving and sustaining good health. In these instances, participants perceive health holistically and beyond the individual level–bringing up the benefits of attending cultural ceremonies, maintaining strong relationships with loved ones, and having a connection to the land.

Holding a close connection to land and the natural environment has been recognized as a significant determinant of Indigenous peoples’ wellbeing, given how important land is in supporting physical, mental, emotional, and spiritual health [27–29, 56, 57]. Communities, as well, have long been understood as a part of Indigenous identities and can play an integral role in healing processes and overall wellbeing [58]. Within our study, participants described how they look out for others and establish security by socially confiding with each other. Social support gained from close relationships or community bonds have been understood to be as important as other more established protective factors for health [26, 59, 60]. Not only are communities able to act as networks for social supports and relevance through shared experiences, they are also sources of strength for Indigenous women to draw on [58]. Individuals can draw strength from their communities through feeling like they belong to a wider collective, but also because engaging within communities is critical to expressing Indigenous identity [58]. In connection to social gatherings, participants described a longing for culture and the importance of cultural activities in promoting mental, emotional, and spiritual health. Cultural activities appeared to act as a facilitator for social gatherings to occur. Social support and social capital have been interlinked with reinforcing cultural identity for Indigenous peoples [25, 61] and participants vocalized this by recounting the opportunities to build their social networks during or after cultural ceremonies and activities. This reciprocity in creating community bonds allows for cyclical and sustained knowledge sharing, which serves to further compound the long-term benefits of both Indigenous individuals and communities’ health [62, 63]. Feeling a sense of community was mentioned as important to their overall wellbeing, and the participants described instances when they would act as both contributors and beneficiaries to creating a sense of community. For example, participants would act as contributors by passing on knowledge to younger generations within the community, and as beneficiaries by attending cultural ceremonies guided by Elders. Such is true that the transmission of traditional knowledge amongst Indigenous communities can occur in both formal and informal contexts, including social encounters, ceremonial practices or other activities within the community [64]. These descriptions by participants might demonstrate that while societal understandings of health are from an individual standpoint, foundational underpinnings of Indigenous wellbeing are constructed through social bonds and cultural continuity, aspects which are not typically considered in current models of healthy provisioning that are accessed by Indigenous women [3, 26, 65].

### Implications for future research

Referencing the FNHA Health and Wellness model while planning culturally relevant health services or an Indigenous healing program is beneficial for prospective service users as it describes determinants of health that exist across different levels of society for every individual. The ability for Indigenous healing programs to take into account the racist, sexist, and colonialist mindsets of society will be crucial towards program success, particularly in the case of Indigenous women, where intersectionality may result in disproportionate burdens of poor health being experienced [66]. This, in turn, may also help to deepen understandings of the socio-environmental complexities impacting health beyond the individual’s control that equally should be addressed in Indigenous women’s circle of care. Continuing to shift the paradigm from individualistic ideologies of healthcare to a more holistic mindset (as presented by the FNHA model) can be significant in changing the ways which Indigenous women perceive self-reliance as the key to health and wellbeing. Further research should investigate how an Indigenous healing program with health and cultural services can benefit the holistic wellbeing of Indigenous women, as well as how it can encourage more women to seek out supports within the community rather than relying on themselves to care for their body, mind, and soul.

### Limitations

Our study employed convenience sampling as the main recruitment strategy, which limits the population reached, as all recruited participants had already been accessing services offered at Elevate NWO and harm reduction sites. Thus, suggestions made by participants on health and cultural services that they would want or need within the Indigenous healing program are not necessarily indicative of the preferences of those who did not previously access the same harm reduction sites or services. This makes it challenging to create a comprehensive healing program offering services that would be culturally relevant and appropriate for a diverse group of Indigenous individuals.

## Conclusion

Considering the unique needs that Indigenous women require in contrast to standard, westernized health services will be critical in ensuring successful healing programs are developed and implemented. Despite the importance of accessing Indigenous healing approaches for Indigenous health being deeply understood and substantially cited by the literature over the past decades [15, 67], Statistics Canada reported that only roughly one third of Indigenous Peoples living in urban areas have access to traditional medicines and healing practices [68]. Aspects of cultural continuity, self-determination, and knowledge transmission have been identified as critical health determinants, yet they remain excluded from major health care reform initiatives in Canada [69]. Allowing Indigenous control and autonomy over the Healing Program will help to establish cultural safety in Indigenous women’s circle of care. Successful Indigenous health strategies must be grounded in factors such as self-determination in order to ensure responsiveness to the unique needs of every community and embed cultural continuity in models of Indigenous health care provisioning [69]. Furthermore, we are hopeful that this Indigenous healing program will support women towards perceiving the importance of social and cultural determinants of health, and that independence and self-care are only one piece towards achieving holistic wellbeing. As such, the Healing Program resulting from this study will be able to act as a facilitator for Indigenous women in accessing culturally relevant health and social services within their community.

## Supporting information

S1 Checklist Inclusivity in global research. (DOCX)

S1 Table Counted quotations of each theme and subtheme. (DOCX)

S2 Table Age of study participants. (DOCX)
