# Supplementary material for: Looking beyond the individual–The importance of accessing health and cultural services for Indigenous women in Thunder Bay, Ontario
Source: PLoS One. 2023 Mar 1;18(3):e0282484. doi: 10.1371/journal.pone.0282484 (PMC9977040; doi:10.1371/journal.pone.0282484)
Supplement: S1 Checklist — (DOCX) [file pone.0282484.s001.docx]

Inclusivity in global research

PLOS’ policy on inclusivity in global research aims to improve transparency in the reporting of research performed outside of researchers’ own country or community and ensures that PLOS publications reporting global research adhere to high standards for research ethics and authorship. Authors of relevant research articles may be asked to complete the questionnaire below, which outlines ethical, cultural, and scientific considerations specific to inclusivity in global research. This questionnaire may be requested when researchers have travelled to a different country to conduct research, if research uses samples collected in another country, research with Indigenous populations or their lands, or if research is on cultural artefacts. Researchers travelling to another country solely to use laboratory equipment will not normally be required to complete the questionnaire. However, the questionnaire can be requested at the journal’s discretion for any submission – if you have been requested to complete this questionnaire by the PLOS journal you submitted to, please do so.

Please complete the questionnaire below and include this as a Supporting Information file with your manuscript. Note that if your paper is accepted for publication, this checklist will be published with your article in the supporting information files. Please ensure that you reference the checklist in the main body of your manuscript. We suggest adding a subsection ‘Inclusivity in global research’ to your Methods section and adding the following sentence: “Additional information regarding the ethical, cultural, and scientific considerations specific to inclusivity in global research is included in the Supporting Information (SX Checklist)”

The questions have been designed to be applicable to a wide range of study types, and there are subsections for both human subjects research and non-human subjects research. If any of the questions are not relevant to your research please mark them as “N/A” as appropriate.

**Ethical considerations, permits and authorship**

*This section is applicable to all research types.*

Provide details as to who granted permissions and/or consent for the study to take place in the Methods section of your manuscript. This should include the names of **all** ethics boards, governmental organizations, community leaders or other bodies that provided approval for the study. If individuals provided approval refer to these people by their role or title but do not list their name(s).

| Reported on page number: 9  Our study received ethics clearance from Women’s College Hospital Research Ethics Board at Women’s College Hospital and the University of Toronto Research Ethics Board in Toronto, Ontario. |
| --- |

If there were any deviations from the study protocol after approval was obtained please provide details of these changes in the Methods section of your manuscript.

| Reported on page number: N/A |
| --- |

Did this study involve local collaborators that are residents of the country where the research was conducted or members of the community studied? If you do not have any authors from said communities, please provide an explanation for this below.

| Reported on page number: 6  This study included local collaborators. The following information has been included in the manuscript:  Five of the 11 co-authors are First Nations (CAS, CJM, LB, MY, MD) with one author being First Nations and French Acadian (ACB). Several of the authors (ET, CAS, CJM, LB, MY, MD, HG, MA) work in and/or lead organizations which provides health, social and cultural services to Indigenous people. Five authors are academic researchers (JCL, ET, CS, CM, ACB) and 6 are community researchers (CAS, MA, LB, MY, MD, HG). Three authors (JCL, CAS, LB) were hired to work on the research project. All authors are experienced in community-based research (CBR). Seven authors (ET, CHS, CAS, CM, LB, MD, HG) call Thunder Bay home and one co-author (MY) is the executive director of an organization serving Indigenous people that has several regional sites including Thunder Bay. |
| --- |

Everyone listed as an author should meet PLOS’ criteria for authorship and all individuals who meet these criteria should be included in the author byline, rather than the acknowledgements. Authorship criteria is based on the International Committee of Medical Journal Editors (ICMJE) Uniform Requirements for Manuscripts Submitted to Biomedical Journals - for further information please see here: <https://journals.plos.org/plosone/s/authorship>.

| Reported on page number: 6  The Contributor Roles Taxonomy (CRediT) methodology has been used to describe the author’s contributions. |
| --- |

**Human subjects research (e.g. health research, medical research, cross-cultural psychology)**

Did you obtain written informed consent from a representative of the local community or region before the research took place? How did you establish who speaks for the community? Details of written informed consent obtained from study participants should be reported separately in the Methods section of your manuscript.

| Reported on page number: 9  Written informed consent from a representative local community or region before the research took place is not applicable in the urban setting this research took place in. Written and verbal consent was obtained from the study participants. The following is included in the text: “Each eligible participant provided both verbal and written informed consent prior to beginning research activities. Verbal consent was obtained using the online platform Zoom or over the phone and written consent was obtained by delivering the consent form through mail, email or during the in-person meeting. Ongoing consent is required by the research ethics board and was obtained verbally throughout the research.” |
| --- |

How did members of the local community provide input on the aims of the research investigation, its methodology, and its anticipated outcome(s)?

| Reported on page number: 5-7, 8  This research was guided by the community partner and stemmed from a previous research study conducted with and for the community. During planning meetings to developing the project for this grant, the community participated in all discussions and provided feedback on the grant and direction to the research activities. Once funding was obtained, funds were sent to the community where they hired local research staff who became part of the research team. Monthly meetings are held to provide study updates, discuss the budget, and plan future activities. This is reflected in the methods – CBR and Research team. |
| --- |

When engaging with the local community, how did you ensure that the informed consent documents and other materials could be understood by local stakeholders?

| The REB provides feedback on the consent form to ensure that it is understood by a lay person. The community partners are experienced in conducting research and provide feedback on all research materials. The local Indigenous research assistant shares the consent form with participants and also reads the consent form to the study participants and answers any questions. The community partners and other Indigenous research team members provide feedback on all study materials and deliverables. |
| --- |

Will the findings of the research be made available in an understandable format to stakeholders in the community where the study was conducted (e.g. via a presentation, summary report, copies of publications, etc.)? Please provide details of how this will be achieved.

| Yes. Several deliverables are being produced by the community partners and specifically the local Elder and Helper (who also works as the research assistant) particularly in phase 2 of the project (e.g., booklet of teachings, reflections for a report or manuscript, photos and notes to create ceremony pamphlets and brochures, and a Faq sheet). During phase 1, the information collected will be used to write manuscripts as a research team and all team members can attend data analysis and writing meetings. They can also lead the development of manuscripts with support from the nominated PI. Abstracts and a presentation or several presentations will also be created that community can deliver. Infographics summarizing manuscripts will be created and shared broadly. Usually, a student research assistant or staff member is assigned to begin the manuscript writing process. They review the transcripts and note the different themes that emerge and the stories that can be told. The emerging themes are shared with the research team which includes community members and meetings are held to further analyze the data and write a manuscript. The nominated PI creates other KTE material that is shared with the research team for feedback. |
| --- |

**Non-human subjects research using specimens/ animals collected as part of the study, or those housed in archival collections. Examples include archaeology, paleontology, botany and zoology.**

Did the permission you obtained from a local authority to perform the study include an agreement on access to outputs and benefit sharing? This may include procedures to enable fair distribution of the benefits and resources arising from the research performed. Please include any details of Prior Informed Consent and Benefit Sharing Agreements obtained. These may be required by field-specific regulations, for example the Convention on Biological Diversity (CBD) and the associated Nagoya Protocol.

| Reported on page number: N/A |
| --- |

If the material used in your study was imported, please A) provide the year it was imported and B) indicate whether permits were obtained to import/export the materials used, C) provide details of any permits obtained. If this information is not available, please indicate this.

| Reported on page number: N/A |
| --- |

If you used archival specimens, please state how the material used in your study was acquired by the institute it is held in and provide details of any permits obtained for the original excavations/ sample collection. If this information is not available, please indicate this.

| Reported on page number: N/A |
| --- |

How was the potential cultural significance of the materials collected in your study to local communities considered in your research design? Were Indigenous peoples and/or local researchers and institutions involved with archaeological excavations / collection of specimens? If so, please provide a description of their involvement.

| Reported on page number: N/A |
| --- |

If your manuscript includes photographs of human remains please indicate whether authors obtained permission from descendants or affiliated cultural communities to do so.

| Reported on page number: N/A |
| --- |
