# Supplementary material for: Looking beyond the individual–The importance of accessing health and cultural services for Indigenous women in Thunder Bay, Ontario
Source: PLoS One. 2023 Mar 1;18(3):e0282484. doi: 10.1371/journal.pone.0282484 (PMC9977040; doi:10.1371/journal.pone.0282484)
Supplement: S1 Table — (DOCX) [file pone.0282484.s002.docx]

**S2 Table. Counted quotations of each theme and subtheme**

| **Theme** | **Subtheme** | **Counted quotations representing theme** |
| --- | --- | --- |
| Independence and self-care | - | 18 |
| External barriers to accessing services | COVID-19 | 12 |
|  | Distance and transportation | 6 |
| Finding comfort in the familiar | Personal relationships | 25 |
|  | Connection to land and nature | 12 |
|  | Demonstrated interest in culturally relevant activities | 24 |
| Sense of community | Knowledge and learning | 16 |
|  | Gathering | 11 |
