# Supplementary material for: Looking beyond the individual–The importance of accessing health and cultural services for Indigenous women in Thunder Bay, Ontario
Source: PLoS One. 2023 Mar 1;18(3):e0282484. doi: 10.1371/journal.pone.0282484 (PMC9977040; doi:10.1371/journal.pone.0282484)
Supplement: S2 Table — (DOCX) [file pone.0282484.s003.docx]

**S3 Table. Age of study participants**

| **What is your age in years?** |
| --- |
| 36 |
| 51 |
| 39 |
| 30 |
| 36 |
| 41 |
| 41 |
| 41 |
| 46 |
| 37 |
| 44 |
| 27 |
| 54 |
| 31 |
| 20 |
| 27 |
| 47 |
| 31 |
| 42 |
| 44 |
| 27 |
| 51 |
| 18 |
| 51 |
| 34 |
| 32 |
| 30 |
| 40 |
| 57 |
| 30 |
| 41 |
| 33 |
| 40 |
| 49 |
